# Whole-genome analysis of a ST45-SCC*mec* IVa (2B)-t116 methicillin-resistant *Staphylococcus aureus* strain isolated from the sputum of a 5-year-old child with pneumonia

**DOI:** 10.3389/fcimb.2024.1413024

**Published:** 2025-01-21

**Authors:** Lin Huang, Rui Guo, Jingxian Lin, Xiaowei Li, Zhicong Li, Limei Zhang, Wenting Li, Rui Xue, Cheng Zhang, Xiaosan Feng, Xiaobin Li

**Affiliations:** ^1^ Department of Pediatrics, Zhuhai People’s Hospital (The Affiliated Hospital of Beijing Institute of Technology, Zhuhai Clinical Medical College of Jinan University), Zhuhai, China; ^2^ Guangdong Provincial Key Laboratory of Tumor Interventional Diagnosis and Treatment, Zhuhai People’s Hospital (The Affiliated Hospital of Beijing Institute of Technology, Zhuhai Clinical Medical College of Jinan University), Zhuhai, China; ^3^ School of Clinical Medicine, Capital Medical University, Beijing, China; ^4^ Department of Pulmonary and Critical Care Medicine, Zhuhai People’s Hospital (The Affiliated Hospital of Beijing Institute of Technology, Zhuhai Clinical Medical College of Jinan University), Zhuhai, China

**Keywords:** methicillin-resistant Staphylococcus aureus (MRSA), ST45, SCC*mec* IVa, prophage, virulence

## Abstract

**Introduction:**

Methicillin-resistant *Staphylococcus aureus* (MRSA) sequence type (ST) 45 is a major global MRSA lineage with huge strain diversity and a high clinical impact. In Hainan and Guangzhou of China, the ST45-MRSA was mainly associated with t116.

**Methods:**

The MRSA strain SA2107 was isolated from the sputum of a 5-year-old child with pneumonia. The whole genome of SA2107 was sequence using Illumina (Novaseq 6000) and PacBio (Sequel IIe) sequencers, and the sequences were assembled using hybrid assembly. The carriage of antibiotic resistance genes, virulence genes, and mobile genetic elements were identified using bioinformatics tools. The comparative genomic analyses of MRSA strain SA2107 with other MRSA strains worldwide were performed.

**Findings:**

The genome size of ST45-SCC*mec* IVa (2B)-t116 MRSA strain SA2107 was ~2.9 Mb. Mobile genetic elements analysis of SA2107 revealed two plasmids (30,064-bp pSA2107-1 and 8,033-bp pSA2107-2), three prophages, two integrative and conjugative elements (ICEs), and two insertion sequences (ISs, IS*431* and IS*1272*). The SCC*mec* IVa (2B) carried by SA2107 contained the class B *mec* gene complex (IS*431*-*mecA*-Δ*mecR1*-IS*1272*) and type 2 *ccr* gene complex (*ccrA2* and *ccrB2*). Besides *mecA*, another beta-lactam resistance gene *blaZ* was found to located on six copies of *bla* complex (*blaZ*, *blaR1*, and *blaI*) on the chromosome of SA2107. Three kinds of virulence factors were detected on the chromosome of SA2107, including genes encoding toxins, exoenzyme, and immune-modulating protein. Notably, the three prophages harbored by the chromosome of SA2107 all carried virulence genes.

**Conclusion:**

Thus far, only three complete genomes available for ST45-SCC*mec* IVa (2B)-t116 strain from United States, Germany, and Australia, respectively. The strain SA2107 was the first complete genome data (CP104559) from China for ST45-SCCmec IVa (2B)-t116 MRSA.

## 1 Introduction

*Staphylococcus aureus*, an important Gram-positive pathogen, can cause various infectious diseases including bacteremia and pneumonia (Bashabsheh et al., 2024). It represents a growing public health burden owing to the emergence and spread of antibiotic-resistant clones, particularly within the hospital environment (Kholaseh et al., 2023). Based on data from the China Antimicrobial Surveillance Network (CHINET), in 2022, *S. aureus* was the third most common clinical bacterial isolate, comprising 9.5% of all clinical bacterial isolates, and it had the highest detection rate in Gram-positive clinical bacteria (https://www.chinets.com/). Methicillin-resistant *S. aureus* (MRSA) is major causative agent for nosocomial infections and has become a difficult problem in treatment of infections (Lee et al., 2018).

*S. aureus* ST45 is a major global MRSA lineage with different regions, hosts, antimicrobial susceptibility, and clinical manifestations, which frequently causes severe invasive disease, such as bacteremia (Effelsberg et al., 2020). *S. aureus* ST45 was originally identified in Berlin in 1993 (Witte et al., 2001) and now widely distributed worldwide (Peng et al., 2024). Epidemiological surveillance has shown that ST45-MRSA was mainly associated with t116 in Hainan and Guangzhou of China, with 70% and 66.7%, respectively (Ge et al., 2019; Li et al., 2019).

The cause of MRSA resistance to beta-lactam antibiotics is *mecA* and its homologues (*mecB*, *mecC*, and *mecD*) carried by MRSA (Lakhundi and Zhang, 2018). The *mec* genes are widely disseminated among staphylococcal species due to the acquisition and insertion of the staphylococcal cassette chromosome *mec* (SCC*mec*) element into the chromosome of susceptible strains, which is responsible for conferring the broad-spectrum beta-lactam resistance (Lakhundi and Zhang, 2018; Uehara, 2022). The SCC*mec* is a mobile genetic element (MGE) composed of *mec* complex, *ccr* complex and J region (Wang et al., 2022b). SCC*mec* elements were classified into different types based on the combination of *mec* gene complex (five classes) (Liu et al., 2016) and *ccr* gene complex (nine classes) (Wu et al., 2015). To date, 15 SCC*mec* types (types I-XV) have been officially reported (Wang et al., 2022b). Notably, subtypes of type IV SCC*mec* vary more than other types, with the main subtypes as follows: IVa, IVb, IVc, IVd, IVg, IVh, IVi, IVj, IVk, IVl, IVm, IVn,and Ivo (Uehara, 2022).

The aim of the study was to report the whole-genome sequence of a MRSA ST45-t116 strain SA2107 harboring SCC*mec* type IVa, which was isolated from the sputum of a 5-year-old child with pneumonia in China.

## 2 Materials and methods

### 2.1 Bacterial isolate and antimicrobial susceptibility testing

This sample was obtained from the sputum of a 5-year-old child with pneumonia at Zhuhai People’s Hospital, Guangdong Province, China. Strain identification was performed using the VITEK-2 Compact system (bioMérieux, France), which was also confirmed by sequencing of the entire 16S rRNA gene. Antimicrobial susceptibility testing was measured by the VITEK 2 COMPACT system, which used the following 15 antimicrobial agents: clindamycin, daptomycin, gentamicin, levofloxacin, moxifloxacin, rifampicin, teicoplanin, vancomycin, ceftaroline, erythromycin, linezolid, oxacillin, penicillin, sulfamethoxazole/trimethoprim, and tigecycline. The results of other antimicrobial agents were interpreted according to the Institute of Clinical and Laboratory Standards (CLSI M100–S33) (CLSI, 2023).

### 2.2 Genome sequencing, assembly, and annotation

Whole-genome sequencing (WGS) of strain SA2107 was conducted by Genewiz Biotechnology Co. Ltd. (Suzhou, China). Genomic DNA was extracted from the strain SA2107 using a genomic DNA extraction kit (provided by Genewiz) according to the manufacturer’s instructions. WGS was performed using paired-end sequencing with Novaseq 6000 (Illumina, 2×150 bp paired-end reads) and long sequencing with PacBio Sequel IIe (Pacific Biosciences, 10-15 Kb insert whole-genome shotgun libraries). PacBio reads were assembled using Hifiasm (version 0.13-r308) (Cheng et al., 2021) and Canu (version 2.2) (Koren et al., 2017). Genome assembly polishing was performed with Pilon (version 1.22) (Walker et al., 2014) using Illumina reads. The assembled genome of *S. aureus* SA2107 was submitted to the NCBI GenBank database (Benson et al., 2018) and annotated using the NCBI Prokaryotic Annotation Pipeline (PGAP) (Tatusova et al., 2016).

### 2.3 Bioinformatics analysis

Acquired antibiotic resistance genes (ARGs) of the genome of SA2107 were identified using ResFinder 4.1 software (Bortolaia et al., 2020), with a minimum identity of 90% and a minimum coverage of 60%. The PointFinder software (Zankari et al., 2017) was used to detect the chromosomal gene mutations mediating antimicrobial resistance. Virulence genes of the genome of SA2107 were identified using VirulenceFinder 2.0 (Joensen et al., 2014). Multilocus sequence typing (MLST) of SA2107 was performed using MLST 2.0 (Larsen et al., 2012). Plasmid replicon types were determined using PlasmidFinder 2.1 (Carattoli et al., 2014). SCC*mec* typing was performed using the web-based SCCmecFinder version 1.2 (Kaya et al., 2018). *spa* typing was performed using the wed-based SpaFinder version 1.0 (Bartels et al., 2014). MGEs of SA2107, including genomic islands and prophage, were identified by the VRprofile2 (Wang et al., 2022a). Sequence similarity searching was performed using MegaBLAST (Morgulis et al., 2008) scans against the GenBank non-redundant (nr) database. Easyfig software (Sullivan et al., 2011) and BLAST Ring Image Generator (BRIG) software (Alikhan et al., 2011) was used to compare and visualize the sequences.

## 3 Results

### 3.1 Identification and antimicrobial susceptibility results of the strain SA2107

The strain SA2107 was identified as *S. aureus* using the VITEK 2 COMPACT system, and BLASTN analysis against rRNA_typestrains/16S_ribosomal_RNA database indicated that the entire 16S rRNA gene of SA2107 showed highest similarity with that of *S. aureus* strain S33 R (GenBank accession NR_037007, 100% coverage and 99.87% identity). Based on the results of the antibiotic susceptibility test, strain SA2107 exhibited resistance to oxacillin, penicillin, clindamycin, and erythromycin as well as intermediate-level resistance to rifampicin (**Supplementary Table S1**).

### 3.2 Genomic characteristics of the strain SA2107

Genome sequencing and analysis revealed that SA2107 contained one 2.83 Mb chromosome (CP104559) and two plasmids with sizes of 30,064 bp (pSA2107-1, CP104560) and 8,033 bp (pSA2107-2, CP104561), respectively. Results of PlasmidFinder indicated that plasmid pSA2107-1 contained two replicons (rep16 and repUS5), whereas the plasmid pSA2107-2 consists of *repB* family plasmid replication initiator (coordinate: 4572.5429). MLST analysis indicated that *S. aureus* strain SA2107 belonged to sequence type (ST) 45. *spa* typing indicated that SA2107 displayed *spa*-type t116.

ResFinder results indicated that SA2107 carried two kinds of beta-lactam resistance genes (*mecA* and *blaZ*), which only located on the chromosome of SA2107, the *mecA* gene is known to confer resistance to methicillin in MRSA isolates and the *blaZ* is the structural gene of the staphylococcal penicillinase. It’s worth noting that SA2107 was found to harbor six copies of *bla* complex (*blaZ*, *blaR1*, and *blaI*) on its chromosome, with coordinates as 283131.286210, 477278.480357, 893462.896541, 2018600.2021679, 2036382.2039461, and 2548907.2551986, respectively. PointFinder results indicated that the rifampin resistance of SA2107 may be due to H481N mutation in RpoB. However, no ARG or chromosomal point mutation linked to clindamycin and erythromycin resistance was found. By the way, PointFinder software also detected many unknown point mutations (**Supplementary Table S2**), some of which might be associated with resistance to clindamycin and erythromycin. VirulenceFinder results indicated that different virulence factors were also detected on the chromosome of SA2107 (**Table 1**), including toxins (*hlgA*, *hlgB*, *hlgC*, *sec*, *seg*, *sei*, *sel*, *sem*, *sen*, *seo*, and *seu*), exoenzyme (*aur*), and genes encoding immune-modulating protein, i.e., hostimm (*sak* and *scn*). However, neither ARG nor virulence gene was detected on the two plasmids of SA2107.

**Table 1:** Virulence genes harbored by the chromosome of SA2107.

Categories	Virulence gene	Position in chromosome	Protein function
Toxin	*sec*	852439.853238	enterotoxin C
	*sel*	853405.854127	enterotoxin L
	*seg*	1871665.1872441	enterotoxin G
	*sen*	1872724.1873500	enterotoxin N
	*seu*	1873518.1874288	enterotoxin U
	*sei*	1874442.1875170	enterotoxin I
	*sem*	1875205.1875924	enterotoxin M
	*seo*	1876205.1876987	enterotoxin O
	*hlgA*	2505651.2506580	gamma-hemolysin chain II precursor
	*hlgC*	2507148.2508095	gamma-hemolysin component C
	*hlgB*	2508097.2509073	gamma-hemolysin component B precursor
Exoenzyme	*aur*	2741397.2742926	aureolysin
Hostimm	*scn*	2058687.2059037	staphylococcal complement inhibitor
	*sak*	2061251.2061742	staphylokinase

Results of VRprofile2 indicated that three prophages, two integrative and conjugative elements (ICEs) and one SCC*mec* were identified on the chromosome of SA2107 (**Table 2**). Notably, the three prophages were found to carry the virulence genes (**Table 2**), indicating their role in the horizontal transfer of these virulence genes.

**Table 2:** MGEs carried by the chromosome of SA2107.

MGE Type	MGE	Coordinate	ARG/Virulence gene (genes)
Prophage	Prophage1	838274.857713	*sec*, *sel*
	Prophage2	1857030.1876986	*seg*, *sen*, *seu*, *sei*, *sem*, *seo*
	Prophage3	2054857.2109023	*scn*, *sak*
ICE	ICE1	1215357.1357201	–
	ICE2	1962688.1985407	–
SCC*mec*	SCC*mec* IVa	39167.54373	*mecA*

ICE, integrative and conjugative element; SCC*mec*, staphylococcal cassette chromosome *mec.*

**Figure 1:**
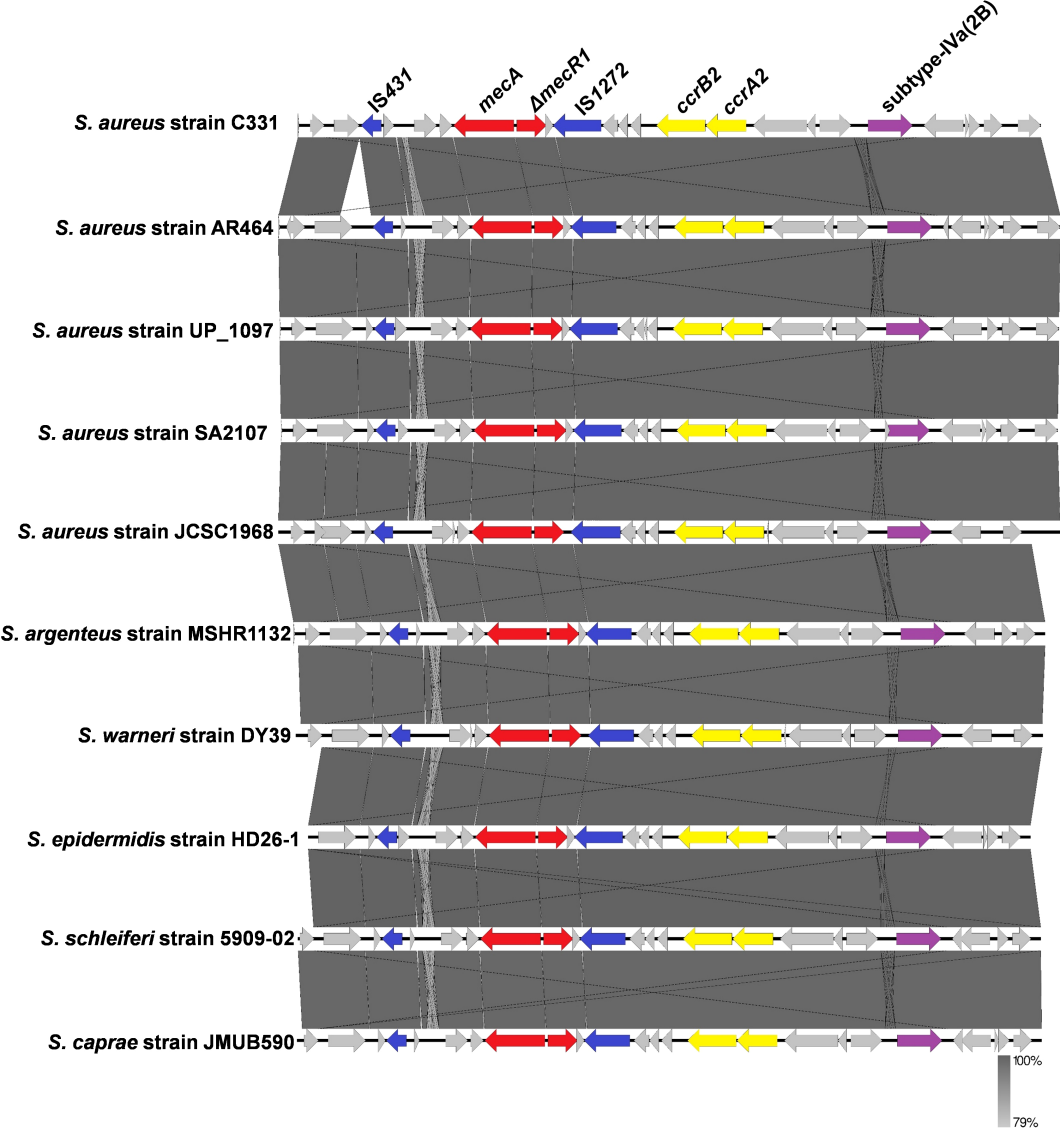
Comparative analysis of the SCC*mec* type IVa (2B) carried by the ST45-t116 MRSA strain SA2107 with other methicillin-resistant staphylococci generated by Easyfig.

Based on the result of SCCmecFinder, the strain SA2107 carried the SCC*mec* type IVa (2B) on its chromosome. The SCC*mec* type IVa (2B) of SA2107 contained the class B *mec* complex, in which *mecA* and the truncated *mecR1* gene encoding the signal transducer protein (Δ*mecR1*) were flanked by IS*431* and IS*1272*, forming the structure IS*431*-*mecA*-Δ*mecR1*-IS*1272* (**Figure 1**). In addition, the type 2 *ccr* gene complex (*ccrA2* and *ccrB2*) was found in the SCC*mec* type IVa (2B) of SA2107 aligned with *S. aureus* JCSC1968, and other staphylococci species reference genomes (**Figure 1**). Notably, based on the results of the BLAST search hit from the nr database of GenBank, the whole SCC*mec* type IVa (2B) detected in strain SA2107 was not only present in the *S. aureus*, but also in the *Staphylococcus caprae*, *Staphylococcus schleiferi*, *Staphylococcus epidermidis*, *Staphylococcus argenteus*, and *Staphylococcus warneri* (**Figure 1**; **Supplementary Table S3**).

### 3.3 An overview of ST45 SCC*mec* type IVa global strains

**Figure 2:**
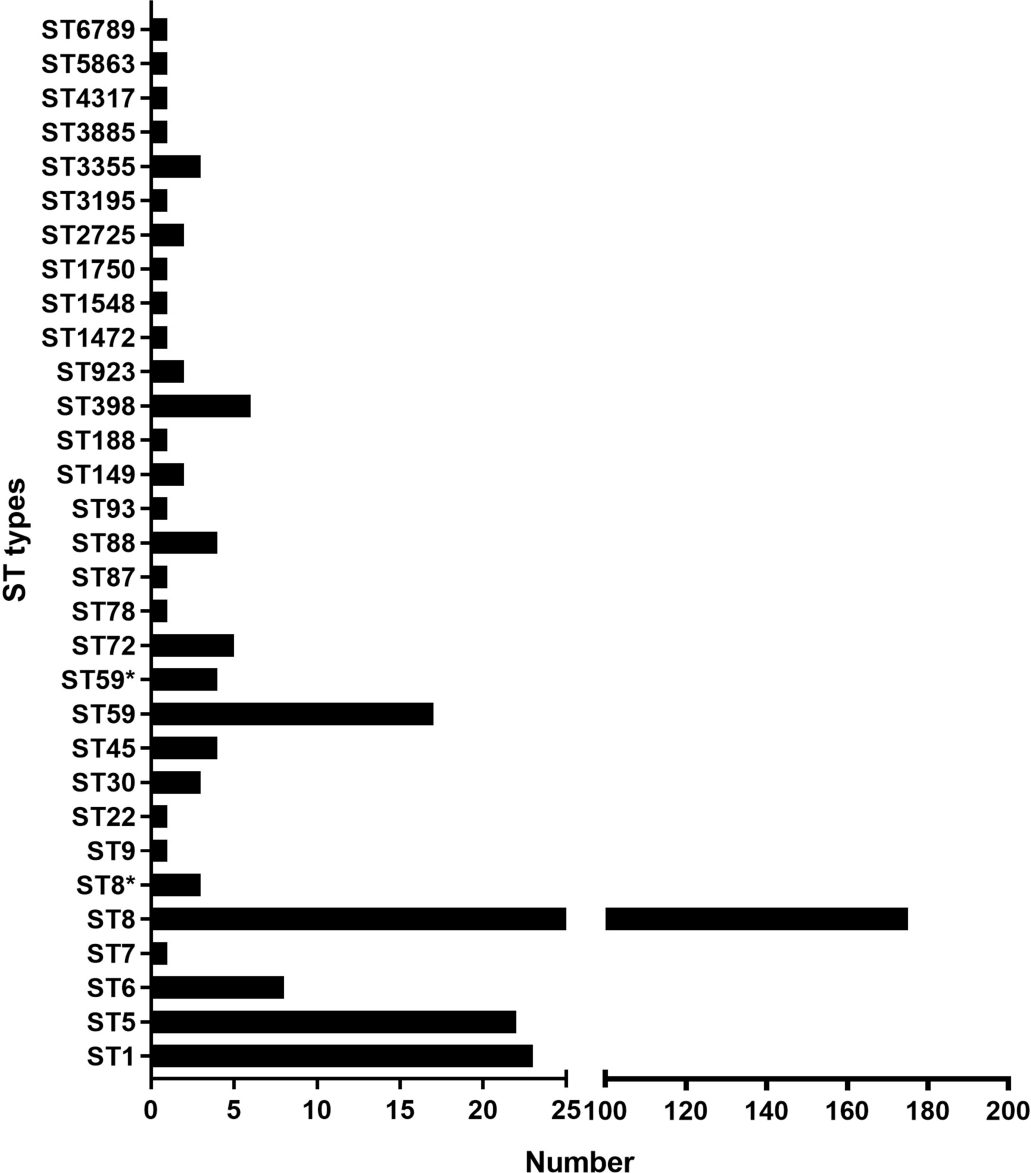
An overview of the ST types among the 301 of MRSA strains harboring the SCC*mec* type IVa. Histogram about number of the SCC*mec* type IVa distributed in different ST types of MRSA was drawn. * alleles with less than 100% identity found.

**Figure 3:**
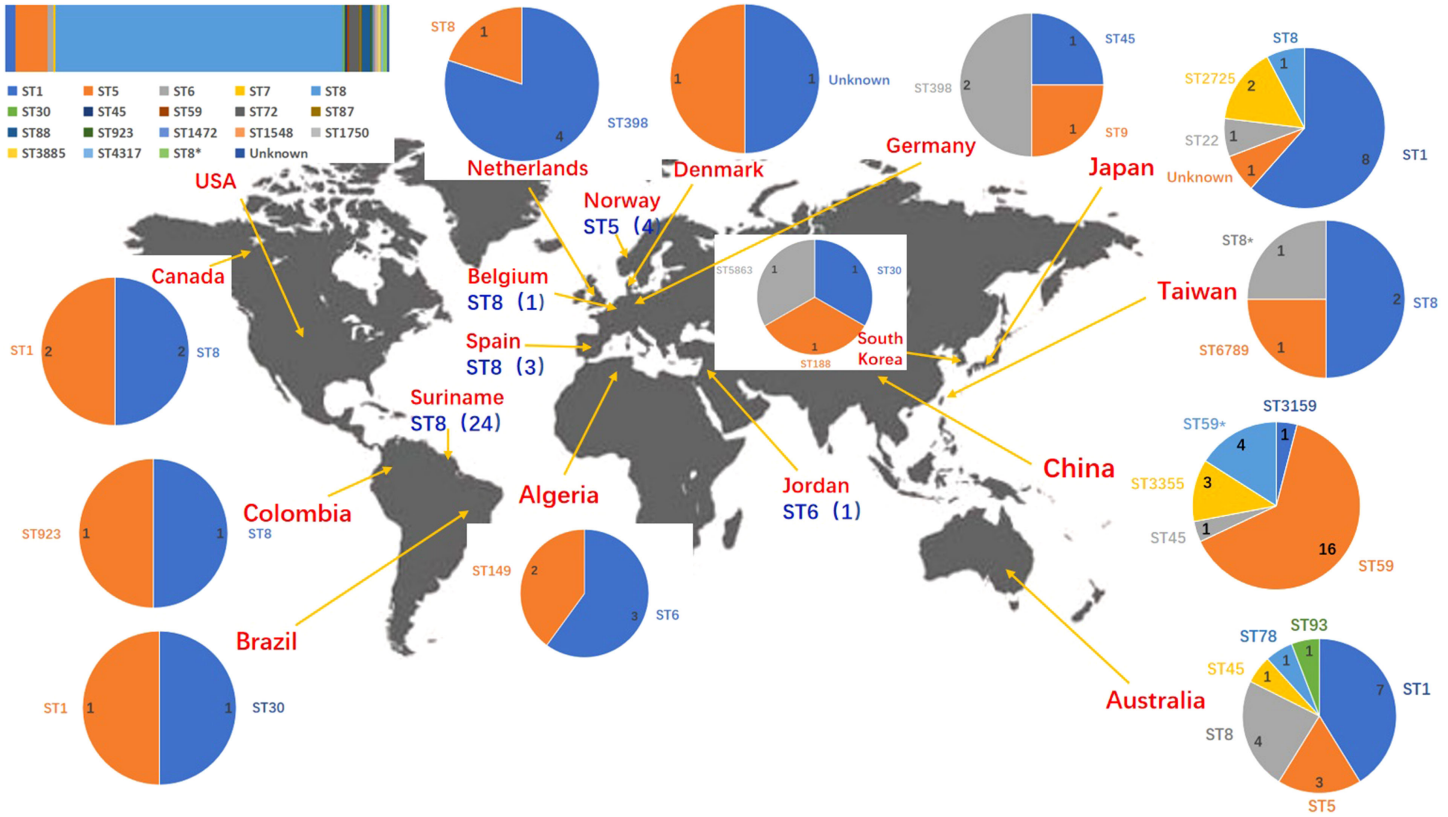
Geographic distribution of the MRSA strains with complete genomes harboring the SCC*mec* type IVa. ST types of different countries/regions were displayed by pie chart except the USA. ST types of the USA were displayed by columnar stacking diagram. * alleles with less than 100% identity found.

**Figure 4:**
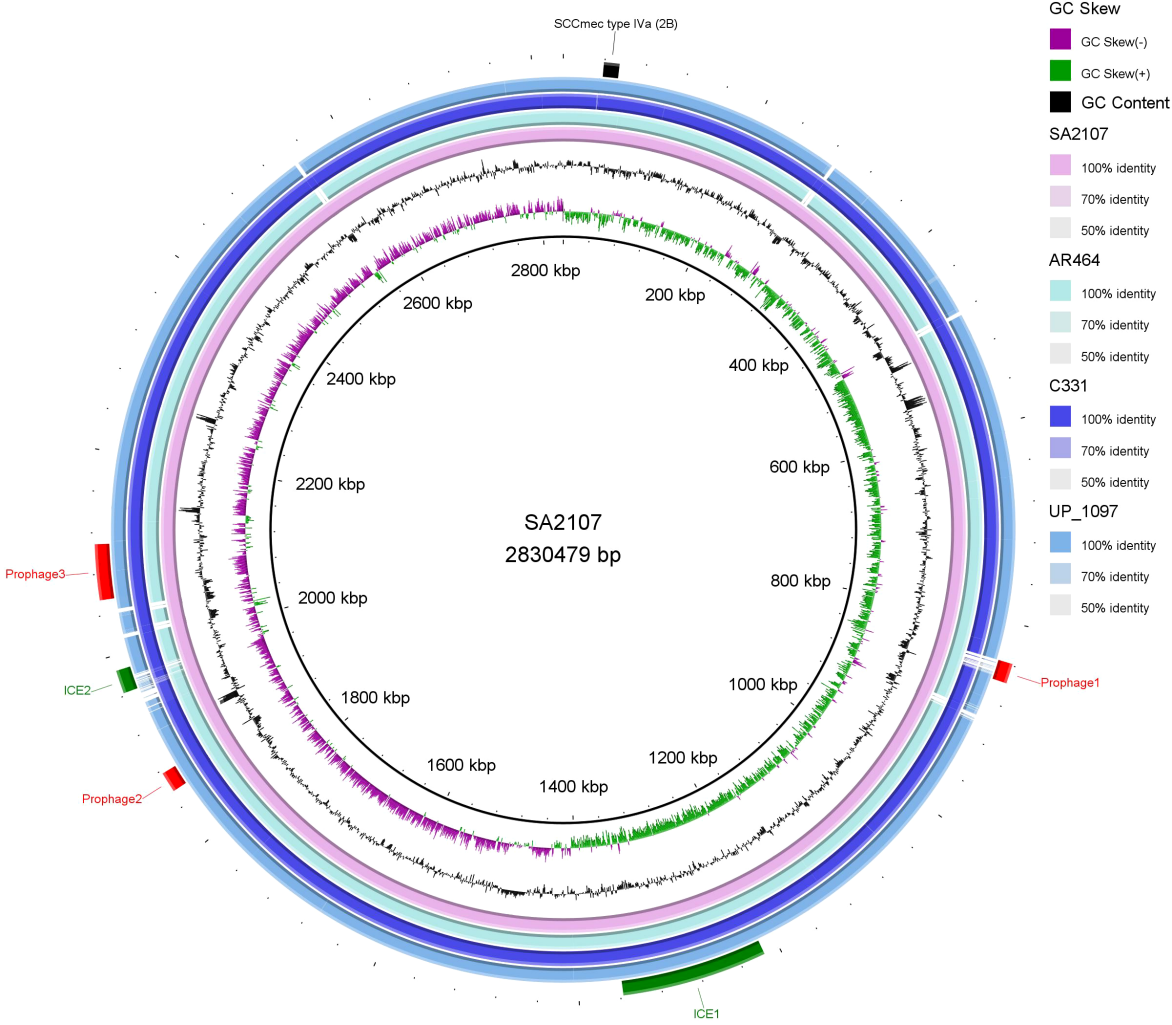
Comparative analysis of the MGEs, including the prophages, ICEs, and SCC*mec* type IVa (2B), carried by SA2107 with other three *S. aureus* ST45 harboring SCC*mec* type IVa (2B) generated by BRIG. Prophages, ICEs, and SCC*mec* are shown in red, green, and black arcs, respectively. GC Skew (+): the relative content of G is greater than C; GC Skew (-): the relative content of G is less than C.

NCBI blast search of SCC*mec* type IVa (2B) of SA2107 against *S. aureus* (taxid: 1280), hit a total of 301 strains of *S. aureus* with complete genomes harboring SCC*mec* type IVa, which was widely present in more than 30 different STs of *S. aureus* (**Figure 2**). The most common ST amongst the SCC*mec* type IVa was ST8 (58.1%, *n*=175), followed by ST1 (7.6%, *n*=23), ST5 (7.3%, *n*=22), and ST59 (5.6%, *n*=17) (**Figure 2**). SA2107 in our study was the first report on the complete genome of the SCC*mec* type IVa (2B) in *S. aureus* ST45 in China (**Figure 3**). Another three strains of *S. aureus* ST45 with complete genomes containing the SCC*mec* type IVa (2B) were found in the United States (CP029084, AR464), Germany (CP047803, UP_1097), and Australia (CP127579, C331), respectively (**Figure 3**). The distribution of MGEs (prophages, ICEs, and SCC*mec* type IVa) carried by SA2107 was explored in other three *S. aureus* ST45 harboring SCC*mec* type IVa (**Figure 4**). Two prophages harboring virulence genes carried by SA2107, prophage2 (*seg*, *sen*, *seu*, *sei*, *sem*, *seo*) and prophage3 (*scn*, *sak*), were found to distributed in all other three ST45-SCC*mec* IVa strains. However, the prophage1 harboring *sec* and *sel* was only found in SA2107 and AR464. In addition, the ICE1 (coordinate: 1215357.1357201) carried by SA2107 was also found to distributed in all other three ST45-SCC*mec* IVa strains.

## 4 Discussion

The ST45-t116 MRSA strain SA2107 was found to contain the SCC*mec* type IVa (2B) on its chromosome. The SCC*mec* type IV has the combination of class B *mec* gene complex and a type 2 *ccr* gene complex (Hiramatsu et al., 2001), just as the “IS*431*-*mecA*-ΔmecR1-IS*1272*” and “*ccrA2* and *ccrB2*” carried by the MRSA strain SA2107 in this study. The SCC*mec* type IVa was first reported in *S. aureus* CA05 (JCSC1968) isolated from the joint fluid of a patient with septic arthritis and osteomyelitis (Ma et al., 2002). Interestingly, only four strains of MRSA ST45-SCC*mec* IVa-t116 with complete genome worldwide harbor SCC*mec* type IVa, and SA2107 is the first report with complete genome in China.

For the SCC*mec* type IVa of MRSA SA2107, the *mecA*-Δ*mecR1* was flanked by IS*431* and IS*1272*. IS*431*, a staphylococcal insertion sequence (IS)-like element related to IS*26* from *Proteus vulgaris*, was first described in 1987, which has been implicated in the transfer of antimicrobial resistance genes (e.g., *mecA* conferring methicillin resistance) (Barberis-Maino et al., 1987; Kobayashi et al., 2001). For the five classes of *mec* gene complexes have been described to date in MRSA (Liu et al., 2016), four were found to contain the IS*431*, including the class A *mec* gene complex (*mecI*-*mecR1*-*mecA*-IS*431*) (Ito et al., 2001; Liu et al., 2016), class B *mec* gene complex (IS*431*-*mecA*-Δ*mecR1*-IS*1272*) (Hiramatsu et al., 2001), the class C *mec* gene complex (IS*431*-*mecA*-*ΔmecR1*-IS*431*) (Katayama et al., 2001) and the class D *mec* gene complex (IS*431*-*mecA*-Δ*mecR*) (Liu et al., 2016). IS*1272* was first described in *Staphylococcus haemolyticus* isolated in the United States in 1996 (Archer et al., 1996), has disseminated to other staphylococcal species and is prevalent in multi-resistant isolates (Wolska-Gębarzewska et al., 2023).

In this study, six copies of *bla* complex (*blaZ*, *blaR1*, and *blaI*) were found on the chromosome of SA2107. The beta-lactamase gene *blaZ* was the structural gene of the staphylococcal penicillinase, and the *bla* complex was necessary for penicillinase production (Massidda et al., 2006). The *blaR1* gene encoded a signal-transducing membrane protein, and the *blaI* gene encoded a repressor protein (Hackbarth and Chambers, 1993). The expression of *blaZ* was regulated by the two adjacent genes, *blaI* and *blaR1*, the first being a *blaZ* transcription repressor, and the second an anti-repressor (Lowy, 2003).

Three categories of virulence genes were identified in the genome of SA2107, including toxin genes, exoenzyme genes, and hostimm genes. Until now, more than 24 staphylococcal enterotoxin (SE) genes have been identified from different outbreaks of staphylococcal food poisoning, clinical cases and strains isolated from animals (Lefebvre et al., 2022; Cieza et al., 2024). Eight SE genes (*sec*, *seg*, *sei*, *sel*, *sem*, *sen*, *seo*, and *seu*) were found in SA2107 and six genes (*seg*, *sei*, *sem*, *sen*, *seo*, and *seu*) were found on prophage2 of the SA2107. Schwendimann et al. reported that these six genes were also found on the *S. aureus* genomic island *v*Saβ (Schwendimann et al., 2021).

## 5 Conclusion

This study describes the genomic characteristics of a ST45-t116 MRSA strain SA2107 harboring SCC*mec* type IVa (2B), which is the first complete genome data (CP104559-CP104561) from China for ST45-SCC*mec* IVa (2B)-t116, and can be the reference genome for ST45-SCC*mec* IVa (2B)-t116 MRSA.

## Data Availability

The datasets presented in this study can be found in online repositories. The names of the repository/repositories and accession number(s) can be found below: https://www.ncbi.nlm.nih.gov/genbank/, CP104559–CP104561.
