# Supplementary material for: Whole-genome analysis of a ST45-SCCmec IVa (2B)-t116 methicillin-resistant Staphylococcus aureus strain isolated from the sputum of a 5-year-old child with pneumonia
Source: Front Cell Infect Microbiol. 2025 Jan 21;14:1413024. doi: 10.3389/fcimb.2024.1413024 (PMC11790441; doi:10.3389/fcimb.2024.1413024)
Supplement: Supplementary file 1 [file DataSheet1.docx]

**Whole-genome analysis of** **a** **ST45-****SCC*mec* IVa (2B)-t116** **methicillin-resistant** ***Staphylococcus aureus* strain** **isolated from** **the sputum of a 5-year-old child with pneumonia**

**Table legends**

Table S1. Antibiotic susceptibilities of the SA2107.

Table S2. Uknown point mutations of the chromosome of SA2107 detected by the PointFinder software.

Table S3. Staphylococci species harboring the SCC*mec* type IVa identical to that of SA2107.

Table S1. Antibiotic susceptibilities of the SA2107

| Agents | Abbreviation | MIC (μg/ml) | Interpretation |
| --- | --- | --- | --- |
| Clindamycin | CM | 0.25 | R |
| Daptomycin | DAP | 0.25 | S |
| Gentamicin | GM | ≤0.5 | S |
| Levofloxacin | LEV | ≤0.12 | S |
| Moxifloxacin | MXF | ≤0.25 | S |
| Rifampicin | RA | 2 | I |
| Teicoplanin | TEC | ≤0.5 | S |
| Vancomycin | VA | ≤0.5 | S |
| Ceftaroline | CTR | 0.5 | S |
| Erythromycin | E | ≥8 | R |
| Linezolid | LNZ | 2 | S |
| Oxacillin | OXI | ≥4 | R |
| Penicillin | P | ≥0.5 | R |
| Sulfamethoxazole/Trimethoprim | SXT | ≤10 | S |
| Tigecycline | TGC | ≤0.12 | S |

Note: S, Susceptible; R, Resistant; I, Intermediate

Table S2. Uknown point mutations of the chromosome of SA2107 detected by the PointFinder software.

| Mutation | Nucleotide change |
| --- | --- |
| *23S* |  |
| *23S* r.2797C>T | C -> T |
| *23S* r.410G>A | G -> A |
| *23S* r.1465_1466insG | ins -> G |
| *23S* r.1010G>A | G -> A |
| *23S* r.2320C>T | C -> T |
| *23S* r.1010G>A | G -> A |
| *23S* r.2320C>T | C -> T |
| *23S* r.67G>A | G -> A |
| *23S* r.1262T>C | T -> C |
| *23S* r.1901C>T | C -> T |
| *23S* r.67G>A | G -> A |
| *23S* r.595G>A | G -> A |
| *23S* r.853G>T | G -> T |
| *dfrB* |  |
| *dfrB* p.V72E | GTG -> GAG |
| *dfrB* p.V76A | GTA -> GCA |
| *dfrB* p.I97T | ATA -> ACA |
| *grlA* |  |
| *grlA* p.S267G | AGT -> GGT |
| *grlA* p.F594Y | TTT -> TAT |
| *grlA* p.A688V | GCT -> GTT |
| *grlA* p.V694M | GTG -> ATG |
| *grlB* |  |
| *grlB* p.E422D | GAA -> GAT |
| *grlB* p.D530G | GAT -> GGT |
| *grlB* p.E596D | GAA -> GAC |
| *gyrA* |  |
| *gyrA* p.V248E | GTT -> GAA |
| *gyrA* p.V598I | GTC -> ATC |
| *gyrA* p.S668A | TCA -> GCG |
| *gyrA* p.V712I | GTT -> ATT |
| *gyrA* p.T825_S826del | ACTTCA -> ------ |
| *gyrA* p.R837H | CGT -> CAT |
| *gyrA* p.D856E | GAT -> GAA |
| *gyrA* p.N860T | AAT -> ACT |
| *ileS* |  |
| *ileS* p.N213D | AAC -> GAT |
| *ileS* p.A223S | GCA -> TCA |
| *ileS* p.N257D | AAT -> GAT |
| *ileS* p.E259Q | GAA -> CAA |
| *ileS* p.I263V | ATT -> GTT |
| *ileS* p.D276G | GAT -> GGC |
| *ileS* p.A280D | GCA -> GAT |
| *ileS* p.Y288F | TAC -> TTT |
| *ileS* p.T289S | ACA -> TCA |
| *ileS* p.E306K | GAA -> AAA |
| *ileS* p.D313E | GAT -> GAA |
| *ileS* p.Q421K | CAA -> AAA |
| *ileS* p.E472D | GAA -> GAT |
| *ileS* p.S570A | TCA -> GCT |
| *ileS* p.T814S | ACT -> AGT |
| *ileS* p.D866E | GAT -> GAA |
| *ileS* p.V877A | GTC -> GCC |
| *pbp2* |  |
| *pbp2* p.E269Q | GAA -> CAA |
| *pbp2* p.A285P | GCT -> CCT |
| *pbp2* p.E315A | GAA -> GCA |
| *pbp2* p.A576S | GCT -> TCT |
| *pbp2* p.A606D | GCA -> GAC |
| *pbp4* |  |
| *pbp4* p.T25A | ACT -> GCT |
| *pbp4* p.Y208F | TAT -> TTT |
| *pbp4* p.L234H | CTA -> CAT |
| *pbp4* p.V381F | GTT -> TTT |
| *pbp4* p.E398A | GAA -> GCA |
| *pbp4* p.T409A | ACA -> GCA |
| *pbp4* p.R430I | AGA -> ATA |
| *rpoB* |  |
| *rpoB* p.D320N | GAT -> AAT |
| *pbp4*-promoter |  |
| *pbp4*-promoter n.-54A>G | A -> G |
| *pbp4*-promoter n.-62G>T | G -> T |
| *pbp4*-promoter n.-160A>G | A -> G |
| *pbp4*-promoter p.T25A | ACT -> GCT |

Table S3. Staphylococci species harboring the SCC*mec* type IVa identical to that of SA2107

| Strain | GenBank accession | Coverage | Identity |
| --- | --- | --- | --- |
| *S. aureus* strain C331 | CP127579 | 95% | >99% |
| *S. aureus* strain AR464 | CP029084 | 100% | >99% |
| *S. aureus* strain UP_1097 | CP047803 | 100% | >99% |
| *S. aureus* strain JCSC1968 | AB063172 | 100% | >99% |
| *S. caprae* JMUB590 | AP018586 | 95% | >99% |
| *S. schleiferi* 5909-02 | CP009676 | 95% | >99% |
| *S. epidermidis* HD26-1 | CP052998 | 92% | >99% |
| *S. argenteus* MSHR1132 | FR821777 | 96% | >99% |
| *S. warneri* strain DY39 | KU170612 | 95% | >99% |
